# Somatic Mutations, Allele Loss, and DNA Methylation of the Cub and Sushi Multiple Domains 1 (CSMD1) Gene Reveals Association with Early Age of Diagnosis in Colorectal Cancer Patients

**DOI:** 10.1371/journal.pone.0058731

**Published:** 2013-03-07

**Authors:** Austin Y. Shull, Megan L. Clendenning, Sampa Ghoshal-Gupta, Christopher L. Farrell, Hima V. Vangapandu, Larry Dudas, Brent J. Wilkerson, Phillip J. Buckhaults

**Affiliations:** 1 Georgia Health Sciences University Cancer Center, Georgia Health Sciences University, Augusta, Georgia, United States of America; 2 Department of Biochemistry and Cancer Biology, Georgia Health Sciences University, Augusta, Georgia, United States of America; 3 Department of Pharmaceutical and Administrative Sciences, Presbyterian College School of Pharmacy, Clinton, South Carolina, United States of America; 4 Graduate School of Biomedical Sciences, The University of Texas M. D. Anderson Cancer Center, Houston, Texas, United States of America; 5 Northeast Georgia Medical Center, Department of Internal Medicine, Gainesville, Georgia, United States of America; 6 Department of Otolaryngology-Head and Neck Surgery, University of California Davis Medical Center, Sacramento, California, United States of America; University of Illinois at Chicago, United States of America

## Abstract

**Background:**

The Cub and Sushi Multiple Domains 1 (CSMD1) gene, located on the short arm of chromosome 8, codes for a type I transmembrane protein whose function is currently unknown. CSMD1 expression is frequently lost in many epithelial cancers. Our goal was to characterize the relationships between CSMD1 somatic mutations, allele imbalance, DNA methylation, and the clinical characteristics in colorectal cancer patients.

**Methods:**

We sequenced the CSMD1 coding regions in 54 colorectal tumors using the 454FLX pyrosequencing platform to interrogate 72 amplicons covering the entire coding sequence. We used heterozygous SNP allele ratios at multiple CSMD1 loci to determine allelic balance and infer loss of heterozygosity. Finally, we performed methylation-specific PCR on 76 colorectal tumors to determine DNA methylation status for CSMD1 and known methylation targets ALX4, RUNX3, NEUROG1, and CDKN2A.

**Results:**

Using 454FLX sequencing and confirming with Sanger sequencing, 16 CSMD1 somatic mutations were identified in 6 of the 54 colorectal tumors (11%). The nonsynonymous to synonymous mutation ratio of the 16 somatic mutations was 15∶1, a ratio significantly higher than the expected 2∶1 ratio (p = 0.014). This ratio indicates a presence of positive selection for mutations in the CSMD1 protein sequence. CSMD1 allelic imbalance was present in 19 of 37 informative cases (56%). Patients with allelic imbalance and CSMD1 mutations were significantly younger (average age, 41 years) than those without somatic mutations (average age, 68 years). The majority of tumors were methylated at one or more CpG loci within the CSMD1 coding sequence, and CSMD1 methylation significantly correlated with two known methylation targets ALX4 and RUNX3. C:G>T:A substitutions were significantly overrepresented (47%), suggesting extensive cytosine methylation predisposing to somatic mutations.

**Conclusions:**

Deep amplicon sequencing and methylation-specific PCR reveal that CSMD1 alterations can correlate with earlier clinical presentation in colorectal tumors, thus further implicating CSMD1 as a tumor suppressor gene.

## Introduction

Colorectal cancer is the third most common cancer with approximately 1 million annual cases worldwide [1]. This complex disorder is normally characterized by an abundance of somatic mutations [2]. However, every tumor has its own unique mutational landscape [3], and the underlying mechanisms that give rise to this diverse landscape are poorly understood. Activating mutations in the adenomatous polyposis coli (APC), Kirsten ras (KRAS), and Phosphatidylinositol 3-kinase (PIK3CA) genes are very common in colorectal cancers [4]. It is likely that the right combination of mutations could give a particular clone of cells a proliferative advantage. Mutations that cause or contribute to tumor progression are commonly referred to as drivers, whereas mutations that offer no selective advantage are commonly referred to as passengers [5], [6], [7]. The majority of somatic mutations that accumulate in tumors are silent passengers [8], [9], whereas small subsets of mutations are actual drivers [10], [11]. By assuming that all silent (synonymous) mutations are passengers, the background rate of somatic mutations in colorectal cancers has been approximated to be 1 mutation per megabase [12]. Genes that are more frequently mutated than the predicted background rate may be drivers of neoplastic development. By assuming that all silent mutations are passengers and that non-silent (nonsynonymous) mutations may either be drivers or passengers, the overall ratio of nonsynonymous to synonymous (NS/S) mutations in a given gene can provide additional evidence as to whether that gene is under positive or negative selection for changes to the amino acid sequence. Genes that accumulate mutations, yet are under no selective pressure, have an expected nonsynonymous to synonymous (NS/S) ratio of approximately 2∶1. This expected ratio is based upon the first two nucleotide positions in the codon dictating the encoded amino acid and the third “wobble” position allowing for codon redundancy. This mechanistic setup of the genetic code creates twice as many opportunities for a given single nucleotide substitution to change the amino acid sequence as there are opportunities to substitute a nucleotide yet preserve the amino acid sequence. Genes with an overrepresentation of non-synonymous mutations have a NS/S ratio that is statistically significantly higher than the expected 2∶1 and can confidently be assumed to be under positive selective pressure to change the amino acid sequence during the process of tumor evolution [13]. Therefore, a high NS/S ratio of somatic mutations can provide statistical evidence to whether a mutated gene is a driver of tumor progression [3], [5].

The Cub and Sushi Multiple Domains 1 (CSMD1) gene is a novel candidate tumor suppressor gene located on the p arm of chromosome 8 (2792875…4852328). CSMD1 is frequently shown to be deleted [14], [15], [16], mutated [2], [10], [17], or methylated [14], [18] in many cancers. In fact, CSMD1 expression is frequently lost in breast cancer [19], whereas CSMD1 loses allelic balance in head and neck squamous cell carcinomas (HNSCC) and lung cancers [20]. CSMD1 has also shown to be methylated in HNSCC cell lines [21].

The first exon of CSMD1 harbors a methylated CpG island (Chromosome 8: 4848969–4852635) [21], a sequence rich in C:G base pairs contained within CpG or CpHpG contexts [22]. The CpG Island Methylator Phenotype (CIMP) [23] describes a subset of colorectal tumors that show high methylation frequency of specific CpG islands. These CIMP tumors have distinct clinical, pathologic, and molecular signatures such as proximal tumor location, poor differentiation, and microsatellite instability. Important clinical correlations with CIMP have also been observed in breast and brain tumors [24] indicating that the phenomenon is not just tissue specific. It is not yet clear if CIMP status causes or is merely associated with these phenotypes. Methylation of specific CIMP genes is only loosely correlated with repression of gene expression; therefore other mechanisms influencing clinical behavior of CIMP tumors may be important. Germline nucleotide substitutions that involve C:G>T:A transitions are thought to be catalyzed by cytosine methylation [25]. These substitutions lead to a genome-wide underrepresentation of CpG dinucleotides compared to what is expected by chance alone. Genome methylation has thus influenced genome sequence evolution and the polymorphism landscape of most vertebrates. By similar reasoning, it is possible that CIMP status of tumor cells or their pre-cancerous precursors (stem cells) may predispose methylated genes to accumulate C:G>T:A somatic mutations.

Somatic mutations in tumors may provide historical evidence for CSMD1 silencing by methylation in cancer progenitor cells. Colon stem cells located at the base of the crypts may be the primary targets for malignant transformation [26], [27]. Mutations that originate in stem cells [28], [29], [30], [31] have an opportunity to persist long enough for the accumulation of cooperating oncogenic mutations. Although epigenetic silencing of CSMD1 may contribute to the malignant transformed phenotype, [32], [33], [34], [35], [36], [37], somatic mutations that accumulate as a result of methylation may also contribute to malignancy by unknown mechanisms. By examining multiple methods of CSMD1 alterations (mutation, methylation, and allele loss), we observed significant correlations of CSMD1 loss of function with early age of diagnosis in colorectal cancer patients. The correlation between CSMD1 loss of function and clinical presentation may help provide insight into the neoplastic development of colorectal cancer.

## Materials and Methods

### Tumor Selection and DNA Isolation

De-identified surgical specimens were obtained from the South Carolina Biorepository System (Palmetto Health, Richland; Palmetto Health, Baptist, and Lexington Regional Medical Center, Lexington, South Carolina). This study was conducted with the approval of the Institutional Review Board of Palmetto Health, Lexington Medical Center, University of South Carolina, and Georgia Health Sciences University, and all written patient consents were obtained prior to study. De-identified clinical data was obtained from the tumor-registry, and the manual curation of pathology records was conducted by tissue bank personnel.

We chose 54 microsatellite-stable colorectal tumors for this study. All surgical specimens were fresh-frozen, embedded in Optimal Cutting Temperature compound (Sakura Finetek, Torrence, CA), and sectioned. The samples were cut into 10μm thick slices and fixed onto Sigma silane-prep ä slides. The slides were fixed using 75%, 95%, and 100% ethanol and xylene. The slices from the beginning and end of each tissue sample were stained with Mayer's Hematoxylin (Sigma, St. Louis, MO) and Eosin (Harleco, Lawrence, KS) solutions to specifically obtain the boundaries of tumor tissues. All patient sample data are provided in Table 1.

**Table 1:** Clinical and genetic data of 54 colorectal tumors sequenced by 454FLX sequencing.

Patient	Gender	Race	Age at Diagnosis	Stage	KRAS AA Change	KRAS Somatic%	Allelic Imbalance	CSMD1 Somatic Mutation	CSMD1 Somatic %	CSMD1 Methylation
**510**	F	AA	68	1	WT	0%	Unbalanced		0%	
**517**	M	EA	52	2A	WT	0%	Unbalanced	yes	47%	yes
**529**	F	EA	52	2A	G12>D	32%	Unbalanced	yes	31%	yes
**535**	F	EA	80	1	G12>D	47%	Balanced		0%	yes
**541**	F	EA	84	3B	G13>D	68%	Balanced		0%	yes
**562**	F	EA	69	3B	WT	0%	Unbalanced		0%	
**575**	F	EA	73	3B	G12>V	27%	Balanced		0%	
**587**	M	EA	75	3B	WT	0%	Balanced	yes	46%	yes
**590**	F	EA	69	2A	WT	0%	Indeterminant		0%	yes
**591**	M	EA	75	1	G12>D	68%	Unbalanced		0%	yes
**5102**	M	EA	72	2A	WT	0%	Balanced		0%	
**5143**	M	EA	79	1	WT	0%	Indeterminant		0%	yes
**5155**	F	EA	74	1	WT	0%	Unbalanced		0%	
**5156**	F	EA	61	3B	G13>D	35%	Balanced		0%	
**5161**	F	unk	82	2A	WT	0%	Balanced		0%	yes
**5166**	M	EA	62	4	WT	0%	Balanced		0%	
**5176**	F	EA	55	3C	WT	0%	Indeterminant		0%	yes
**5179**	F	AA	89	2A	WT	0%	Indeterminant		0%	yes
**5190**	M	EA	56	1	G12>V	17%	Balanced		0%	yes
**5191**	M	EA	53	1	WT	0%	Indeterminant		0%	
**10158**	F	EA	76	4	WT	0%	Indeterminant		0%	yes
**14000**	M	AA	73	3B	G12>R	24%	Balanced		0%	yes
**31380**	M	AA	48	2A	WT	0%	Indeterminant		0%	yes
**40099**	F	AA	61	4	WT	0%	Balanced		0%	
**40131**	F	EA	63	3B	G12>D	50%	Unbalanced		0%	
**40195**	M	EA	60	2A	WT	0%	Indeterminant		0%	
**40395**	M	EA	64	1	G12>D	15%	Balanced	yes	33%	
**40415**	M	EA	19	1	WT	0%	Unbalanced	yes	35%	
**40417**	F	EA	80	3A	G12>A	25%	Unbalanced		0%	yes
**40530**	M	EA	71	3B	WT	0%	Balanced	yes	49%	
**40532**	M	EA	81	3B	WT	0%	Indeterminant		0%	yes
**40599**	F	AA	68	3B	WT	0%	Unbalanced		0%	
**40651**	F	AA	30	3B	G12>D	42%	Unbalanced		0%	yes
**40686**	F	AA	73	3A	WT	0%	Indeterminant		0%	yes
**40694**	M	KA	59	4	WT	0%	Unbalanced		0%	
**41043**	M	EA	78	2A	WT	0%	Balanced		0%	
**41044**	F	AA	66	3B	G12>D	61%	Indeterminant		0%	yes
**41065**	F	AA	59	3B	G12>V	16%	Indeterminant		0%	yes
**41084**	F	EA	58	2A	WT	0%	Unbalanced		0%	
**41091**	M	EA	81	3B	WT	0%	Indeterminant		0%	yes
**41098**	F	AA	69	3B	G12>V	49%	Unbalanced		0%	yes
**41104**	F	EA	59	4	G12>V	34%	Balanced		0%	
**41126**	M	EA	57	1	WT	0%	Unbalanced		0%	
**41127**	M	AI	84	2A	WT	0%	Indeterminant		0%	
**41132**	M	AA	63	3A	G13>D	43%	Unbalanced		0%	yes
**41136**	M	EA	68	2A	WT	0%	Unbalanced		0%	
**41141**	F	AA	58	4	G12>D	75%	Indeterminant		0%	
**41142**	F	EA	58	3A	G12>D	18%	Balanced		0%	yes
**41149**	F	EA	59	1	G12>C	24%	Balanced		0%	
**41150**	F	EA	49	2B	WT	0%	Unbalanced		0%	
**41162**	M	EA	64	3C	WT	0%	Indeterminant		0%	yes
**41163**	F	EA	52	1	G12>R	47%	Indeterminant		0%	yes
**41180**	M	EA	64	ND	WT	0%	Unbalanced		0%	
**41185**	F	EA	77	3A	WT	0%	Balanced		0%	

Extractions of the tumor and normal epithelial cells from the tissue slices were performed using micro-dissection technique developed in our lab. The tumor and normal cells were identified for micro-dissection with the use of two prepared H&E slides. A pathologist was involved in the process of identification of tumor and normal tissues. Tumor epithelium was microdissected from sections to obtain approximately 100 μg of DNA. The genomic DNA was then isolated using DNAdvance kit (Agencourt, Beverly, MA).

### Quantification of Genomic DNA

The quantification of the isolated genomic DNA was performed with a standard curve of a known concentration of human DNA and real-time polymerase chain reaction (PCR) primers for Long Interspersed Nuclear Element (LINE) sequences [58]. The primers consisted of a LINE (F) Forward – AAAGCCGCTCAACTACATGG and a LINE (R) Reverse – CTCTATTTCCTTCAGTTCTGCTC (Integrated DNA Technologies, Coralville, IA). Quantification of the DNA was prepared using 6.25 μl of iTaq SYBR green SuperMix (Bio-Rad, Hercules, CA), 1.25 μl of 2 μM LINE F, 1.25 μl of 2 μM LINE R, 2.5 μl of PCR water (Invitrogen, Carlsbad, CA), and 1.25 μl of DNA template. Amplification of the DNA was carried out through thermal cycling with the MyIQ Thermal iCycler (Bio-Rad, Hercules, CA) using the following protocol: denaturation at 95°C for 1 min; 60 cycles at 94°C for 10 sec, 62°C for 45 sec, and 62°C for 5 min. The final template concentrations of tumor and normal DNA were 3ng/μl.

### Screening for Microsatellite Instability in Tumors

The detection of mismatch repair deficient tumors was performed through amplification of microsatellite locus, so that tumors showing hypermutator phenotype could be excluded. All tumors were initially pre-screened for microsatellite instability (MSI) using BAT26 primers, and those with instability were excluded. Subsequently, tumors with somatic mutations to CSMD1 were analyzed with three additional NCI MSI loci: BAT25, D2S123 and D17S250 primers. The primers used are listed in Table S1 in File S1 (Integrated DNA Technologies, Coralville, IA). None of the tumors showed instability at the BAT26 locus, and only one sample (Tumor ID 587) showed moderate microsatellite instability at BAT25 locus. The PCR amplicons were generated with the following protocol: PCR master mix was prepared by using 5 μl of KAPA 5x HiFi buffer (KAPA Biosystems Woburn, MA), 0.75 μl of 10 mM dNTPs, 3.00 μl of primers (final concentration 2.5 μM), 0.50 μl of KAPA HiFi HotStart polymerase (KAPA Biosystems Woburn, MA), 1.00 μl of 10X SYBR Green (Invitrogen, Carlsbad, CA), 8.75 μl of PCR Water (Invitrogen, Carlsbad, CA), and 1 μl of DNA template at 3ng/μl. Thermal cycling was performed on a MyIQ Thermal iCycler (Bio-Rad, Hercules, CA) using the following protocol: 1 cycle of 95°C for 2 min; 3 cycles of 63°C for 15 sec and 72°C for 15 sec; 3 cycles 98°C for 20 sec, 60°C for 15 sec, 72°C for 15 sec; 3 cycles of 98°C for 20 sec, 57°C for 15 sec, 72°C for 15 sec; 49 cycles of 98°C for 20 sec, 56°C for 15 sec, 72°C for 15 sec; 1 cycle 55°C for 30 sec; 80 cycles of 55°C for 30 sec. The PCR products were run on 10% TBE-Urea gels (BIO-RAD, California, USA) according to the manufacturers guidelines and photographed using an Alpha Imager and Quantity One™ software (Alpha Innotech, San Leandro, CA).

### Preparation of Amplicons for Sequencing

The exons for CSMD1 and KRAS genes were identified with the Sequence Viewer on the NCBI Website (http://www.ncbi.nlm.nih.gov/projects/sviewer). The PCR primers were designed for the 71 exons of CSMD1 and for 2 common hotspot mutations in KRAS (exon 2, codon 12 and 13) using the PRIMER3 open source software (http://frodo.wi.mit.edu/primer3/input.htm; (Table S1 in File S1). KRAS was sequenced only at the mutation hotspots due to sample constraints. Forward and reverse tag sequences, 454A and 454B, respectively were added to the primers as described in the Guide to Amplicon Sequencing (454 Life Sciences, Branford CT). PCR amplicons for 454 sequencing were generated using 7.5 μl of KAPA 5x HiFi buffer (KAPA Biosystems Woburn, MA), 1.125 μl of 10mM dNTPs, 2.25 μl of primers (final concentration 2.5 μM), 0.75 μl of KAPA HiFi HotStart polymerase (KAPA Biosystems Woburn, MA), and 1 μl of DNA template at 5ng/μl. Thermal cycling was performed using a MyIQ Thermal Cycler (Bio-Rad, Hercules CA) and the following touchdown protocol: One cycle, 95°C for 2 min; 3 cycles of 94°C for 10 sec, 64°C for 10 sec, 70°C for 30 sec; 3 cycles of 94°C for 10 sec, 61°C for 10 sec, 70°C for 30 sec; 3 cycles of 94°C for 10 sec, 58°C for 10 sec, 70°C for 30 sec; 50 cycles of 94°C for 10 sec, 57°C for 10 sec, 70°C for 30 sec. The PCR products were analyzed by electrophoresis on a 3% agarose gel and photographed using an Alpha Imager and Quantity One™ software (Alpha Innotech, San Leandro, CA). Amplicons were purified using SPRI Ampure beads (Agencourt, Beverly, MA) following the manufacturers protocol.

### Sequencing with 454FLX Platform

The SPRI-Ampure bead purified PCR products (CSMD1 and KRAS) were quantified using the Quanti-iT PicoGreen dsDNA Assay (Invitrogen, Carlsbad, CA) and Fluoroskan Ascent FL *(*Thermo Fisher Scientific Inc., Waltham, MA). The amplicons were amplified by emulsion PCR using the emPCR Kite II and III (Roche Diagnostics, Indianapolis, IN) and bi-directionally sequenced on the 454 GS FLX genome sequencer (University of South Carolina Environmental Genomics Core Facility, Columbia, SC). Amplicons from each of the 54 tumors, as well as 2 paired normal samples, were sequenced to an average depth of 1800 fold with over 90% of the amplicons represented by greater than 300 sequence reads per sample. Over 500,000 individual sequencing reads were obtained from the amplicons.

### Variant Detection Analysis

The 454FLX sequencing reads were aligned to the reference sequence from NCBI (hg19 Build 37) and analyzed using the software CLC Genomics Workbench 4 (CLC Bio, Aarhus, Denmark). We analyzed our sequencing data derived from colorectal tumors and matched normal samples by using the Probabilistic Variant Detection Tool provided by the Genomics Workbench. Mutations seen in the complementary normal tissue, the dbSNP database, or the 1000 Genomes Project were considered to be germline mutations. The remaining mutations were considered as likely somatic mutations. Our mutation calls were made under robust and stringent sequencing criteria set by the CLC Genomics Workbench with a probability call of 100 in order to delete any false mutation observations. Any homopolymer tracts were also excluded from our data analysis. We also excluded discovered insertion/deletion (INDEL) variants from our study, since 454FLX sequencing is not sensitive in detecting INDEL's.

### Somatic Mutation Validation through Sanger Sequencing

Sanger sequencing of the high-concentration variants was performed in order to confirm the somatic mutations identified by the variant detection analysis. Beckman Coulter/Agencourt Genomic Services (Beverly, MA) and the core genomics facility at Georgia Health Sciences University performed the Sanger sequencing, utilizing the same 454A and B tags previously described with the 454FLX sequencing. The PCR amplicons were generated with the following protocol: PCR master mix was prepared by using 7 μl of PCR water (Invitrogen, Carlsbad, CA), 10 μl of 2X KAPA SYBR (KAPA Biosystems Woburn, MA), 2 μl of Primer mix (final concentration 2.5 μM), and 1 μl of DNA template at 3ng/μl. Thermal cycling was performed with the following protocol: 1 cycle of 98°C for 2 min; 3 cycles of 98°C for 10 sec, 63°C for 10 sec, 70°C for 30 sec; 3 cycles of 98°C for 10 sec, 60°C for 10 sec, 70°C for 30 sec; 3 cycles of 98°C for 10 sec, 57°C for 10 sec, 70°C for 30 sec; 49 cycles of 98°C for 10 sec, 56°C for 10 sec, 70°C for 30 sec; 1 cycle of 55°C for 30 sec; 80 cycles of 55°C for 80 cycles. Sanger chromatograms were analyzed using CLC Genomics Workbench 4. After being validated by Sanger sequencing, the CSMD1 somatic mutations were recorded and deposited in the Leiden Open Variation Database (LOVD) (Leiden University Medical Center, Netherlands).

### Allelic Balance Testing from 454FLX CSMD1 Sequences

We used the Sequential Probability Ratio Test (SPRT) to detect statistically significant allelic imbalances in the tumor samples [39], [40]. The SPRT was designed to test two competing hypothesis using sequentially accrued observations over time against pre-specified upper and lower bounds of data [59]. Hypothesis 1 is that the alleles are balanced, so the expected allelic proportions at informative loci should be 50%. Hypothesis 2 is that one of the two alleles present in the normal sample is completely absent in the tumor, so the expected dominant allelic proportion at informative loci should be 100% for tumor samples uncontaminated with normal DNA. In cases undergoing LOH where the tumor DNA is contaminated with up to 50% normal DNA, the expected dominant allelic proportion is 66.7%. Confidence interval curves were constructed to correctly identify balanced and unbalanced alleles across the range of total allele counts observed in the 454FLX data, allowing for up to 50% contamination of tumor with normal DNA. The upper curve establishes the threshold for allelic imbalance, whereas the lower curve establishes the threshold for allelic balance. If a tumor's allele proportion exceeds the upper bound the tumor was classified as unbalanced. In contrast, if the allele proportion is less than the lower bound curve, the tumor was classified as balanced. If the observed allele proportion occurred between the two curves, the samples were classified as indeterminate. In all cases, two or more informative alleles were required to be out of balance to call a tumor imbalanced.

### CSMD1 mRNA Expression Analysis in HCT116 WT and HCT116 DNMT1/DNMT3B DKO

Expression of CSMD1 mRNA was determined by performing quantitative real-time PCR on HCT116 WT and HCT116 DNMT1/3B DKO cDNA. HCT116 WT and DNMT1/3B DKO cDNA was generated by first isolating the mRNA of each using Dynabeads mRNA isolation kit (Invitrogen, Carlsbad, CA) and then was converted into cDNA using Superscript II Reverse Transcriptase (Invitrogen, Carlsbad, CA). The PCR amplicons were generated with the following protocol: PCR master mix was prepared by using 3 μl of PCR water (Invitrogen, Carlsbad, CA), 10μl of 2X KAPA SYBR (KAPA Biosystems Woburn, MA), 2 μl of Primer mix (final concentration 2.5 μM), and 5 μl of cDNA template. Thermal cycling was performed with the following protocol: 1 cycle of 98°C for 2 min; 3 cycles of 98°C for 10 sec, 64°C for 10 sec, 70°C for 30 sec; 3 cycles of 98°C for 10 sec, 61°C for 10 sec, 70°C for 30 sec; 3 cycles of 98°C for 10 sec, 58°C for 10 sec, 70°C for 30 sec; 40 cycles of 98°C for 10 sec, 57°C for 10 sec, 70°C for 30 sec; 1 cycle of 95°C for 30 sec; 80 cycles of touchdown gradient starting at 95°C and decreasing by −5°C each cycle.

### Methylation Analysis using Methylation Specific PCR/High Resolution Melt Curve Analysis

We purified DNA from tumors and control cell lines by the Agencourt DNAdvance Kit (Beckman Coulter Genomics, Beverly, MA) according to manufacturer's instructions. Two hundred nanograms of genomic DNA was bisulfite modified by the EZ DNA Methylation – Gold Kit (Zymo Research), following the manufacturer's protocol. HCT116 and the HCT116-DNMT1/3B double knockout (DKO) DNA [43] were used as positive and negative controls for the methylation status of all genes examined. CSMD1 is methylated and not expressed in the wild-type HCT-116 cells. In contrast, CSMD1 is unmethylated and expressed in the DKO cells [60]. CSMD1 gene methylation was queried at 3 positions and were named CSMD1-1 (Chr8: 4852196–4852032), CSMD1-3 (4851450–4851301) and CSMD1-5 (4851422–4851291). The methylation specific PCR primers used are shown in Table S2 in File S1. Each tumor generated an authentic product either with methylated primers or with unmethylated primers, but never both. Instances where no product was observed with either primer pair were considered uninformative. The methylation results of the tumors are shown in Table S3 in File S1.

The methylation specific PCR/high resolution melt Curve Analysis was implemented according to previously described protocols [61]. The PCR reaction was performed using 3–6 ng of bisulfite modified DNA in a total volume of 20 μl, containing 12.5 μl of 2X KAPA SYBR FAST qPCR Master Mix (KAPA Biosystems Woburn, MA), (for analyzing genomic position 3265577 KAPA HRM fast PCR kit was used); and 2.5pmol of primers [62]. The reaction cycling conditions were 94°C for 10 min, followed by 45 cycles of 30 sec at 94°C, Gene-specific Annealing Temperature for 30 sec, extension at 72°C for 30 sec, and a data collection window at 76–77°C for 30 sec. Association curves were generated for all products, and the presence or absence of authentic products was determined by comparison to positive and negative controls. All reactions were performed using a MyIQ Single color Real-Time PCR Detection System (Bio-Rad).

### Statistical Analysis

Binomial probability was used to compare the significance between nonsynonymous to synonymous somatic mutations of CSMD1 in colorectal patients. Comparison of average age of diagnosis or clinical presentation for genetically balanced and unbalanced patients with somatic mutations were done by the Mann-Whitney U test. Spearman Rank Correlation was used to compare the relationships between methylated loci.

## Results

### Frequencies and Character of Somatic Mutations

The CSMD1 gene spans 2.05 MB of genomic DNA on the p arm of chromosome 8. The coding sequence portion of the gene is 11,740 nucleotides (0.0234 Mb per diploid genome). We sequenced a total of 54 colorectal tumors equaling to 1.26 MB of CSMD1 CDS DNA. We identified and Sanger-verified 16 somatic mutations in 6 of the 54 colorectal tumors (11%) (Table 2). We then calculated a somatic mutation rate of 11.90 mutations/MB, a rate that is noticeably higher than the genome-wide background mutation rates of microsatellite-stable colorectal cancers [3], [38].

**Table 2:** Sanger-validated somatic mutations of CSMD1 in colorectal tumors.

Patient	Chr	Position	Reference	Variants	Counts	Coverage	Frequencies	CDS	AA Change	Conservation score
**517**	8	2813254	G	G/A	696/327	1090	63.9/30.0	9851C>T	Ala3284Val	0.666
**517**	8	2823344	C	C/T	941/357	1425	66.0/25.1	9233G>A	Arg3078His	0.221
**517**	8	2824162	C	C/A	454/172	686	66.2/25.1	9030G>T	Lys3010Asn	0.932
**517**	8	3141735	T	T/C	828/331	1159	71.4/28.6	4084A>G	Asn1362Asp	1
**517**	8	3141850	G	G/A	721/393	1159	62.2/33.9	3969C>T		0.934
**517**	8	3216691	G	G/C	638/486	1161	55.0/41.9	3287C>G	Pro1096Arg	1
**517**	8	3265577	C	C/T	127/58	187	67.9/31.0	1915G>A	Ala639Thr	0.073
**517**	8	3265665	G	G/T	71/29	101	70.3/28.7	1827C>A	Asn609Lys	1
**517**	8	3889468	C	C/T	594/187	856	69.4/21.8	569G>A	Gly190Glu	0.983
**517**	8	4851922	C	C/A	31/28	61	50.8/45.9	17G>T	Arg6Ile	0.296
**529**	8	2967782	A	A/G	157/69	226	69.5/30.5	6506T>C	Leu2169Pro	0.906
**587**	8	3165911	G	G/T	510/192	703	72.5/27.3	3746C>A	Ala1249Asp	1
**40395**	8	3046419	G	G/A	76/38	118	64.4/32.2	5513C>T	Ser1838Leu	0.866
**40415**	8	2944669	C	C/T	165/54	227	72.7/23.8	7424G>A	Arg2475Gln	0.282
**40415**	8	3265619	T	T/G	57/30	89	64.0/33.7	1873A>C	Ile625Leu	0.998
**40530**	8	3267040	G	G/A	196/189	385	50.9/49.1	1649C>T	Thr550Ile	0.984

**Figure 1:**
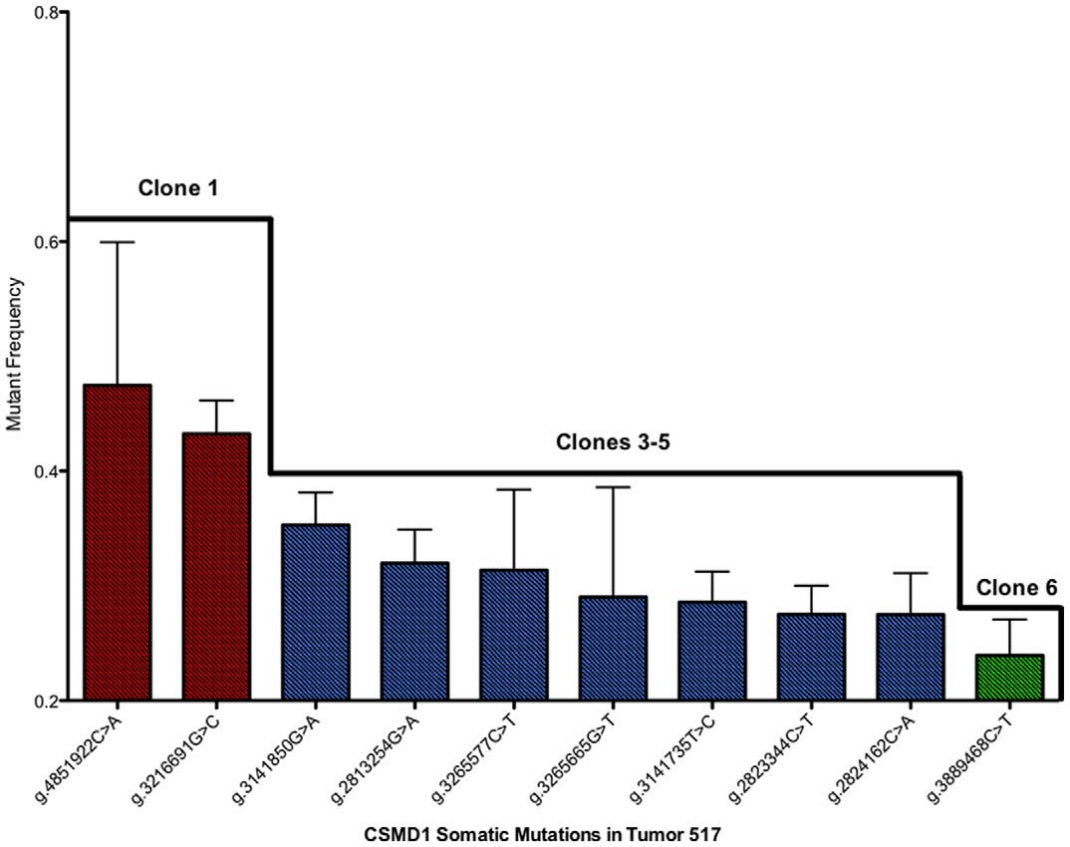
CSMD1 mutation frequencies of somatic variants in Tumor 517. Ten CSMD1 somatic mutation were found in Tumor 517. However, these particular mutations occur at varying subsets of concentrations, indicating that each subset may have arisen from an independent clone within the tumor population.

The somatic mutations were significantly enriched for nonsynonymous changes compared to synonymous changes. For the Sanger confirmed variants, we identified 15 nonsynonymous (NS) mutations and 1 synonymous (S) mutation, for a NS/S ratio of 15∶1. The probability of this many (or more) nonsynonymous mutations occurring by chance alone is 0.014, arguing against the hypothesis that CSMD1 is a passenger gene affected by a mutator phenotype. The more likely explanation is that nonsynonymous mutations are selected for during colorectal cancer development. It is important to note that tumor 517 had 10 somatic mutations to CSMD1, nine of which were nonsynonymous. The probability of this many nonsynonymous mutations occurring by chance alone is 0.1. The alternative hypothesis is that selective pressure drove the accumulation of multiple CSMD1 nonsynonymous variants within the same tumor, possibly residing in separate subclones. The varying subsets of mutation frequencies in CSMD1 allude to this belief that the multiple CSMD1 mutations arose from separate subclones in the same tumor population (Figure 1).

Since progression of adenocarcinoma can be driven by mutations to the KRAS oncogene, we analyzed the mutation hotspots at codons 12 and 13 of exon 2 using Sanger sequencing of PCR products. KRAS hotspot somatic mutations were seen in 21 of the 54 colorectal tumors (∼39%). Three tumors contained somatic mutations in both CSMD1 and KRAS, with two of the three tumors showing a higher CSMD1 mutant allele frequency than the KRAS mutant allele (Table 1). This result may indicate that the CSMD1 somatic mutations predate KRAS somatic mutations, which are thought to occur early during colorectal tumor progression. One tumor did have CSMD1 mutation allele concentrations that were less than the KRAS mutant allele concentration, perhaps indicating that the CSMD1 mutations in this tumor occurred after the KRAS mutations. Still, the clone harboring the CSMD1 mutant allele expanded sufficiently to allow the CSMD1 mutant allele to be detected, demonstrating that this expansion was not driven by the mutant KRAS allele. In order to properly determine the timelines for CSMD1 and KRAS mutations, carful sequence analysis of early lesions such as adenomas will be required. Nevertheless, our observations indicate that CSMD1 nonsynonymous mutations provide tumor cells with a selective advantage, which operates independently of the advantage provided by KRAS mutations.

### Colorectal Tumors with CSMD1 LOH and Somatic Mutations Show Early Clinical Presentation

**Figure 2:**
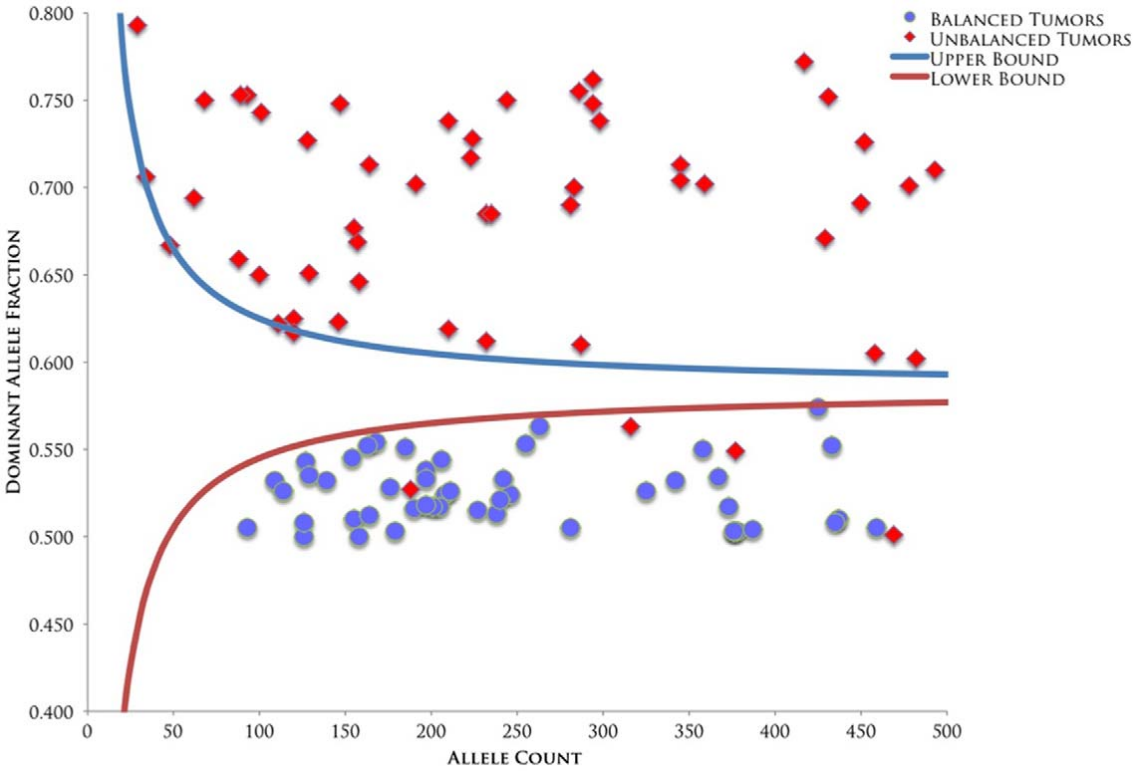
Sequential Probability Ratio Test (SPRT) analysis of CSMD1 based on digital allele counting. The upper bound curve shown in the graph demonstrates the 95% CI threshold of CSMD1 loci being classified as unbalanced, whereas the lower bound curve demonstrates the 95% CI threshold of CSMD1 loci being classified as balanced. Tumors with two or more contiguous loci that were imbalanced were classified as having undergone loss of heterozygosity. Loci that fell between the two thresholds were deemed indeterminate for allelic characterization. Certain loci in CSMD1 demonstrated allelic balance, though they resided in tumors that were unbalanced for CSMD1. This result alludes to chromosomal breaks that occur within the CSMD1 gene sequence, downstream of the examined loci.

Out of the 54 tumors sequenced, Thirty-seven had two or more loci that were heterozygous for germline variants and could therefore be assessed for CSMD1 allelic imbalance by the Sequential Probability Ratio Test [39], [40]. Fifty-one percent (19/37) of the colorectal tumors were unbalanced at two or more contiguous CSMD1 loci (Figure 2, Table 1), indicating that CSMD1 loss of heterozygosity could have occurred in these tumors. Although the difference was not statistically significant, the average age of diagnosis for patients with CSMD1 allelic imbalance was younger than those with balanced alleles (60 years vs. 69 years, Mann-Whitney p = 0.052). The average age at diagnosis for patients with CSMD1 somatic mutations was also younger than for those without somatic mutations (58 years vs. 66 years), though this difference was also not statistically significant. Loss of heterozygosity is a mechanism that cooperates with somatic mutation to inactivate tumor suppressor genes. We observed that patients with both unbalanced and mutated CSMD1 alleles were significantly younger at age of diagnosis (41 years) than patients with balanced and wild type CSMD1 (68 years, Mann-Whitney p = 0.0077). In contrast to our earlier studies [17], we did not observe a significant relationship between CSMD1 somatic mutation status and tumor stage in this series of samples. Nevertheless, the statistical evidence regarding clinical presentation coincides with recent studies conducted by The Cancer Genome Atlas (TCGA). Based on a Kaplan-Meier Survival Analysis, TCGA sequencing data revealed that colorectal cancer patients with CSMD1 alterations have a significantly lower probability of survival than patients without CSMD1 alterations (log-rank test p = .000565) [41]. With such evidence, the identification of CSMD1 alterations has the potential to be used as markers in colorectal cancer prognosis.

### Excess of CG>TA Mutations and CSMD1 Methylation

**Figure 3:**
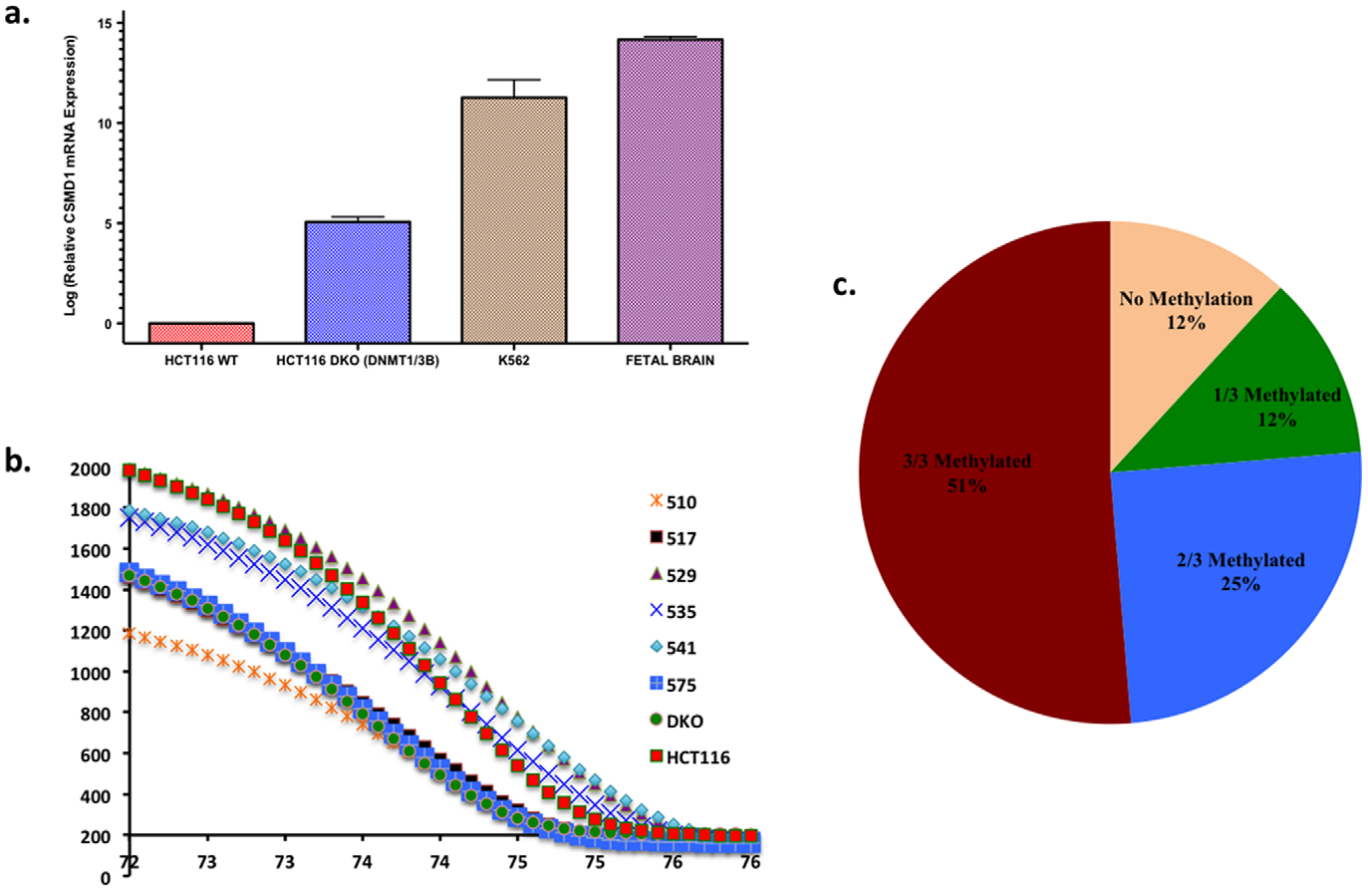
Determining CSMD1 methylation in colorectal tumors. a.) CSMD1 mRNA expression comparison in HCT116 WT and HCT116 DNMT1/3B DKO cDNA along with K562 and fetal brain cDNA. b.) The high-resolution melt curve analysis distinguishes between methylated and unmethylated CG at Reference position 3265577 based upon their melt temperature. Tumor 517 was mutated at CG3265577 so the melt peak coincided with HCT-DKO whereas methylated tumors coincided with the HCT-116 peaks, which is our positive control c.) The percentage of colorectal tumors methylated at CSMD1 loci determined by methylation specific PCR melt curve analysis.

Exome sequencing of colorectal cancers has revealed that several tumors have global excess of CG>TA somatic mutations [2], [3]. We observed 7 out of 15 (46.7%) CSMD1 somatic mutations were CG>TA transitions (Table 2). This class of mutation arises from non-enzymatic deamination of 5′-methylcytosine to produce thymidine. Constitutively methylated CpG dinucleotides have been shown to be mutation hotspots in the retinoblastoma gene (RB1) [42]. Of the CG>TA somatic mutations, 4 were C>T and 3 were G>A (which is assumed to have arisen by C>T on the opposing strand). The even distribution of CG>TA mutations between the transcribed and non-transcribed strands of the CSMD1 gene argues against active transcription-coupled repair occurring during the accumulation of the mutations. A simple explanation for this result could be that the mutations to CSMD1 occurred in a cell type in which the gene is silenced through DNA methylation. By performing quantitative RT-PCR on HCT116 wildtype and HCT116 DNA methyltransferase 1/DNA methyltransferase 3B double-knockout (DNMT1/3B DKO) cell line [43] cDNA (as well as K562 cell line and fetal brain cDNA), we were able to demonstrate that over a 30 fold increase in CSMD1 mRNA expression in HCT116 DNMT1/3B DKO cells compared to their wildtype counterparts. This increase in mRNA expression indicates that DNA methylation, a mechanism depleted by the knockout of both DNMT1 and DNMT3B in HCT116 cells, has an inhibitory effect on CSMD1 expression (Figure 3a). Based on the results of the RT-PCR assay, we wanted to understand the potential correlation between DNA methylation and CG>TA mutations in CSMD1. To do this, we analyzed the genomic position 3265577, which contained a somatic mutation in a C:G context (Tumor 517) and is a part of two neighboring CpG sites (Chr8:3265572-3265582, sequence GAC**CGCG**AGAA). We designed PCR primers specific for bisulfite converted sequences flanking the core CGCG (Table S2 in File S1) and confirmed whether this site is methylated in non-mutated tumors by performing a high-resolution melt association curve analysis (Figure 3b) (Table S3 in File S1). HCT116 WT and HCT116 DNMT1/3B DNA were used as positive and negative controls, respectively, to determine DNA methylation in each tumor sample.

We then analyzed the publicly available data reported by Kim et al. [44] in GSE17648 and found CSMD1 hypermethylation at CG22619018 in the colorectal tumors compared to the adjacent patient-matched normal colonic mucosa (paired t-test, p = 3.59E-08). Although it is impossible to query the methylation status of CSMD1 within the stem cells that gave rise to the tumors, we obtained direct evidence that CSMD1 remains heavily methylated in developed colorectal tumors. We performed methylation-specific PCR on three CSMD1 loci (Chr8: 4852196–4852032; Chr8: 4851450–4851301; Chr8: 4851422–4851291) (Figure S1a & b) and four known CpG Island Methylator Phenotype (CIMP) genes: ALX4 (Chr11: 44282278–44331716), RUNX3 (Chr1: 25226002–25291501), NEUROG1 (Chr5: 134,870741–134,872051) and CDKN2A (Chr9: 21967751–21975132). Remarkably, 90% of tumors examined were methylated in at least one of these three CSMD1 loci, and 50% of tumors were methylated at all three loci (Figure 3c). The frequency of CSMD1 methylation exceeded that of the established CIMP markers. Methylation at all three CSMD1 loci was also significantly positively correlated with RUNX3, whereas CSMD1-3 also significantly correlated with ALX4 (p = 0.001, R = .4206). Although NEUROG1 and CDKN2A have shown to be methylated in colorectal cancers [45], [46], CSMD1-3 methylation did not correlate with NEUROG1 (p = 0.432, R = .1081) or CDKN2A (p = 0.537, R = .08488) methylation, though these two markers did positively correlate with each other (p<0.01) (Table 3 & Table 4). Interestingly, the data from Ben-Porath et al [47] showed that ALX4 and CSMD1 are bound by members of the Polycomb Repressor Complex 2 (PRC2), a complex that silences gene expression through histone modification and predisposes genes to DNA methylation. Our data also show that CSMD1 DNA methylation is significantly positively correlated with both methylated ALX4 and the known PRC2 target RUNX3. Observing RUNX3 methylation in our data coincides with others who have reported that 21% of colorectal cancer have RUNX3 hypermethylation [48]. We conclude that CSMD1 silencing by PRC2-mediated histone modifications may have impacted the somatic mutation landscape through acquired DNA methylation [49], [50].

**Table 3:** Spearman Rank correlation p values of CSMD1 loci analyzed for DNA methylation.

	CSMD1-3	CSMD1-5	ALX4	RUNX3	NEUROG1	CDKN2A
CSMD1-1	**0.001**	**0.001**	0.593	**0.05**	0.614	0.348
CSMD1-3		**0.001**	**0.001**	**0.001**	0.432	0.537
CSMD1-5			0.179	**0.015**	0.681	0.284
ALX4				**0.001**	**0.03**	**0.006**
RUNX3					**0.03**	**0.006**
NEUROG1						**0.001**

**Table 4:** Spearman Rank R values of CSMD1 loci analyzed for DNA methylation.

	CSMD1-3	CSMD1-5	ALX4	RUNX3	NEUROG1	CDKN2A
CSMD1-1	**0.4135**	**0.4292**	0.06131	**0.2141**	0.05794	0.1078
CSMD1-3		**0.3654**	**0.4206**	**0.546**	0.1081	0.08488
CSMD1-5			0.1538	**0.2728**	0.04725	0.1226
ALX4				**0.491**	**0.2362**	**0.3066**
RUNX3					**0.2371**	**0.3077**
NEUROG1						**0.4693**

These results document that several CpG islands within CSMD1 are indeed methylated within tumors. The excess of CG>TA somatic mutations may indicate that the methylation de-stabilized the CSMD1 coding sequence, predisposing it to CG>TA transition mutations. This speculation is supported by the observation that genomic position 3265577 is methylated in colorectal tumors, and was a site of somatic mutation in Sample 517.

## Discussion

We previously reported frequent mutations of CSMD1 in colorectal cancers [17]. In this follow-up study, we used next generation sequencing to further confirm the important role of CSMD1 mutations in colorectal cancer progression. We observed that the CSMD1 somatic mutations are enriched for nonsynonymous changes. We interpret this to indicate that CSMD1 mutation provides a proliferative advantage, even in tumors that have mutations in strong driver genes such as KRAS, PIK3CA, and APC. Functional studies of CSMD1 will be required to further understand the mechanism by which CSMD1 mutations offer a selected advantage during tumor formation. However, the high NS/S ratio of CSMD1 mutations alone highlights the importance of this gene during colorectal tumor development. This high NS/S ratio in CSMD1 mutant alleles (15∶1) approaching clonal dominance is consistent with the Darwinian model of tumor sub-clone development and positive Darwinian selection for nonsynonymous mutations within the primary tumor. Tumor suppressor genes are inactivated in cancer by deletion, mutation, or, in some cases, DNA methylation. Interestingly, some tumor suppressor genes that normally are seen methylated can also be somatically mutated [51]. Our data suggests that CSMD1 is a tumor suppressor gene that can be silenced by multiple mechanisms. CSMD1 variant allele sampling by deep sequencing may be an attractive method for identifying early-stage neoplasia. Variants that lead to frame shifts, nonsense, or non-conservative missense mutations may represent novel reporters foreshadowing poor clinical outcome. Molenaar et al., [52] recently reported recurrent mutations in CSMD1 in neuroblastoma tumors and demonstrated that other genes involved in neuronal cone growths are silenced in neuroblastoma.

Within the somatic mutations discovered, we found a high prevalence of CG>TA mutations, a well as frequent DNA methylation. Cancer-specific hypermethylation of CSMD1 or other methylation markers could result from a particular mutational mechanism. A recent study showed that mutations arising from a mutator phenotype have the most influence on cancer development when mutations occur at early stages during carcinogenesis [53]. Nevertheless, though it is difficult to measure the methylation status of normal intestinal stem cells, the CG>TA mutation excess frames a historical record of the methylation environment present in the progenitor population. Whether or not the cancer methylator phenotype itself is encoded by germline or somatic mutation patterns in the tumor may become apparent as additional methylation targets are sequenced for somatic mutations.

It has been shown in hereditary nonpolyposis colorectal cancer (HNPCC) tumors that promoter hypermethylation may be a likely mechanism to accomplish biallelic inactivation. A study by Rai et al., [54] proposed a model where APC controls intestinal cell fate through DNA methylation. APC and retinoic acid downregulates the demethylases, thereby methylating key genes and committing cells to differentiation. Indeed, we discovered that the differentiated tumors were methylated at one or more loci studied.

Aggressive tumors have an embryonic stem cell-like gene expression signature [55]. Colon epithelial cells arise from intestinal stem cells located in the base of intestinal crypts. Each stem cell divides asymmetrically in order to produce a subsequent stem cell and a transitional amplifying cell that migrates up the crypt-to-tip axis and eventually differentiates [27]. Based on this understanding, we postulate that somatic mutations in CSMD1 accumulate in the stem cell compartment and disrupt CSMD1 expression prior to cancer development. This idea of diminished CSMD1 expression has previously been demonstrated in glioblastoma stem cell lines compared to its normal neuronal stem cell counterpart [56]. Thus, these inactivating CSMD1 mutations may possibly reveal their apparent phenotypic influence on tumor pathology only after mutations in critical gatekeeper genes produce a cell population in which the absence of normal CSMD1 creates a pathophysiological effect.

In conclusion, our study indicates that CSMD1 is highly mutated in colorectal cancer with a C:G bias, suggesting that methylation may contribute to the gene-specific hypermutator phenotype. A recent review by Peltomaki [57] stresses Knudson's two hit theory in which the first hit either occurs in somatic cells, leading to sporadic cancer or in the germline, leading to hereditary cancers. However, the second hit is always somatic in nature. The review continues to discuss how DNA methylation may drive somatic events with age progression and cause defectiveness in the bowel mucosa. Kashuba et al. [51] also alludes to this mutator phenotype by discovering multiple mutations in RASSF1A, a tumor suppressor gene that is commonly methylated. Therefore, based on our results and the validation of future studies, CSMD1 methylation may be identified as an instrumental mechanism in driving somatic mutations in CSMD1, indicating itself as an underlying agent in early colorectal cancer development.

## Supporting Information

File S1 **Table S1.** Primer sequences for PCR amplification. **Table** **S2**. List of methylation specific primers. **Table** **S3.** Methylation landscape of CSMD1 studied in tumors.
(XLSX)

Figure S1 **Characterization of CSMD1 methylation in colorectal tumors.** a.) A demonstration of methylation-specific PCR melt curve analysis peaks that differentiate between methylated and unmethylated ALX4 in colorectal cancer tumors studied. b.) A demonstration of methylation-specific PCR melt curve analysis peaks that differentiate between methylated and unmethylated peaks in CSMD1 at position CG3265577 in the colorectal tumors studied.
(TIF)
