# Supplementary figures and images for: Somatic Mutations, Allele Loss, and DNA Methylation of the Cub and Sushi Multiple Domains 1 (CSMD1) Gene Reveals Association with Early Age of Diagnosis in Colorectal Cancer Patients
Source: PLoS One. 2013 Mar 7;8(3):e58731. doi: 10.1371/journal.pone.0058731 (PMC3591376; doi:10.1371/journal.pone.0058731)

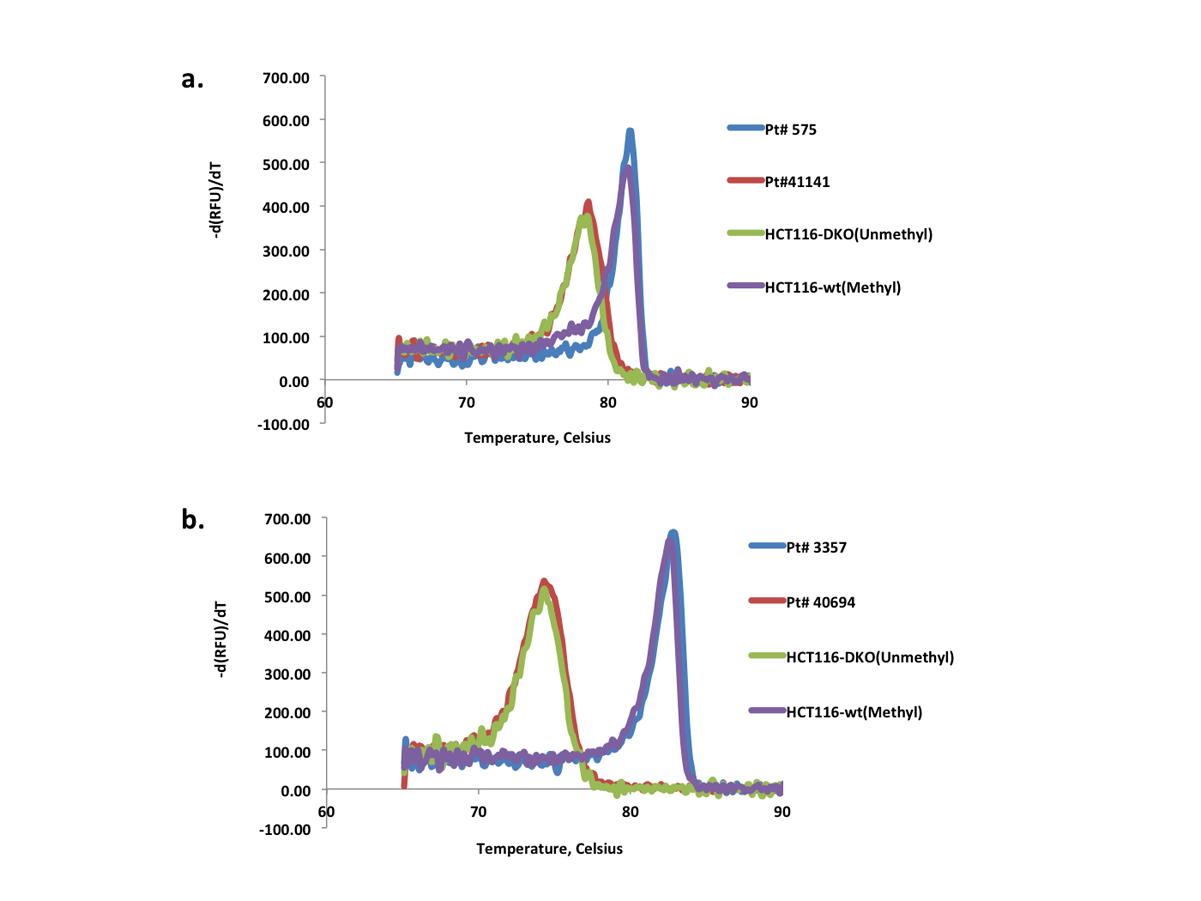

Supplement: Figure S1 — Characterization of CSMD1 methylation in colorectal tumors. a.) A demonstration of methylation-specific PCR melt curve analysis peaks that differentiate between methylated and unmethylated ALX4 in colorectal cancer tumors studied. b.) A demonstration of methylation-specific PCR melt curve analysis peaks that differentiate between methylated and unmethylated peaks in CSMD1 at position CG3265577 in the colorectal tumors studied. (TIF) [file pone.0058731.s002.tif]
